# Development and validation of urinary exosomal microRNA biomarkers for the diagnosis of acute rejection in kidney transplant recipients

**DOI:** 10.3389/fimmu.2023.1190576

**Published:** 2023-05-09

**Authors:** Jung-Woo Seo, Yu Ho Lee, Dong Hyun Tae, Yang Gyun Kim, Ju-Young Moon, Su Woong Jung, Jin Sug Kim, Hyeon Seok Hwang, Kyung-Hwan Jeong, Hye Yun Jeong, So-Young Lee, Byung Ha Chung, Chan-Duck Kim, Jae Berm Park, Junhee Seok, Yeong Hoon Kim, Sang-Ho Lee

**Affiliations:** ^1^Division of Nephrology, Department of Internal Medicine, Kyung Hee University, Seoul, Republic of Korea; ^2^Research Laboratory, Medical Science Institute, Kyung Hee University Hospital at Gangdong, Seoul, Republic of Korea; ^3^Division of Nephrology, Department of Internal Medicine, CHA Bundang Medical Center, CHA University, Seongnam, Republic of Korea; ^4^School of Electrical Engineering, Korea University, Seoul, Republic of Korea; ^5^Research Center, Division of Nephrology, Department of Internal Medicine, Seoul St. Mary’s Hospital, College of Medicine, The Catholic University of Korea, Seoul, Republic of Korea; ^6^Division of Nephrology, Department of Internal Medicine, Kyungpook National University Hospital, Daegu, Republic of Korea; ^7^Department of Surgery, Samsung Medical Center, Seoul, Republic of Korea; ^8^Department of Internal Medicine, Inje University Busan Paik Hospital, Busan, Republic of Korea

**Keywords:** kidney transplantation, acute rejection, urine, exosome, microRNA

## Abstract

**Introduction:**

Acute rejection (AR) continues to be a significant obstacle for short- and long-term graft survival in kidney transplant recipients. Herein, we aimed to examine urinary exosomal microRNAs with the objective of identifying novel biomarkers of AR.

**Materials and methods:**

Candidate microRNAs were selected using NanoString-based urinary exosomal microRNA profiling, meta-analysis of web-based, public microRNA database, and literature review. The expression levels of these selected microRNAs were measured in the urinary exosomes of 108 recipients of the discovery cohort using quantitative real-time polymerase chain reaction (qPCR). Based on the differential microRNA expressions, AR signatures were generated, and their diagnostic powers were determined by assessing the urinary exosomes of 260 recipients in an independent validation cohort.

**Results:**

We identified 29 urinary exosomal microRNAs as candidate biomarkers of AR, of which 7 microRNAs were differentially expressed in recipients with AR, as confirmed by qPCR analysis. A three-microRNA AR signature, composed of hsa-miR-21-5p, hsa-miR-31-5p, and hsa-miR-4532, could discriminate recipients with AR from those maintaining stable graft function (area under the curve [AUC] = 0.85). This signature exhibited a fair discriminative power in the identification of AR in the validation cohort (AUC = 0.77).

**Conclusion:**

We have successfully demonstrated that urinary exosomal microRNA signatures may form potential biomarkers for the diagnosis of AR in kidney transplantation recipients.

## 1 Introduction

Kidney transplantation is the ideal treatment option for patients with end-stage kidney disease. Although renal allograft survival has improved substantially over the past decades, acute rejection (AR) continues to be an important cause of both early and late graft loss (1). Generally, graft rejection is diagnosed using invasive graft biopsy, even though it carries a potential risk of complications. Hence, the development of non-invasive biomarkers can help clinicians to predict the occurrence of AR as well as to improve therapeutic decision-making. To date, numerous, potential, AR-specific biomarkers have been documented, but their applications in clinical practice remains uncertain (2).

Exosomes are specialized subsets of extracellular vesicles, 30 – 100 nm in size; they are derived from the inward budding of endosomal membranes, and they play crucial roles in the cell-to-cell communication system as well as in modulation of immune functions (3–6). All human biofluids, including urine, contain exosomes (7). Therefore, the internal contents of urinary exosomes, namely RNA, DNA, proteins, and lipids, might be an attractive source of non-invasive biomarkers that can help to diagnose various kidney diseases. In fact, there are several previous studies to support this hypothesis, i.e., they have demonstrated significant alterations in the biological characteristics of urinary exosomes in patients with different kidney diseases (8–16).

MicroRNAs are small, non-coding RNA molecules that can modulate the translational activity of target messenger RNAs (mRNAs), thereby playing a vital role in the maintenance of cellular homeostasis (17). Interestingly, they are abundantly present in exosomes, and pathological conditions, including kidney diseases, can alter their expressions significantly (18, 19). Although studies have revealed dysregulated microRNA profiles in the urine cell pellets of kidney transplant recipients with graft rejection (20–22), their expression in urinary exosomes is yet to undergo thorough investigation. Previously, we had demonstrated that BK virus-associated microRNAs are expressed exclusively in urinary exosomes of patients with BK virus-associated nephropathy, thereby suggesting the potential use of urinary exosomal microRNAs as non-invasive biomarkers of pathological conditions in kidney transplant recipients (11, 23).

This study aimed to determine the molecular profiles of urinary exosomal microRNAs in kidney transplant recipients, such that novel biomarkers necessary for non-invasive diagnosis of rejection may be established. We observed that the urinary exosomal microRNA expression was significantly altered in kidney transplant recipients with biopsy-proven AR. These distinctive expression signatures of urinary exosomal microRNAs might be used to distinguish AR from other allograft status. Finally, we validated the diagnostic ability of the urinary exosomal microRNA signatures in an independent cohort of kidney transplant recipients. Overall, our data demonstrate the potential of urinary exosomal microRNAs as novel, non-invasive biomarkers for diagnosing AR after kidney transplantation.

## 2 Materials and methods

### 2.1 Overview of the study design

**Figure 1:**
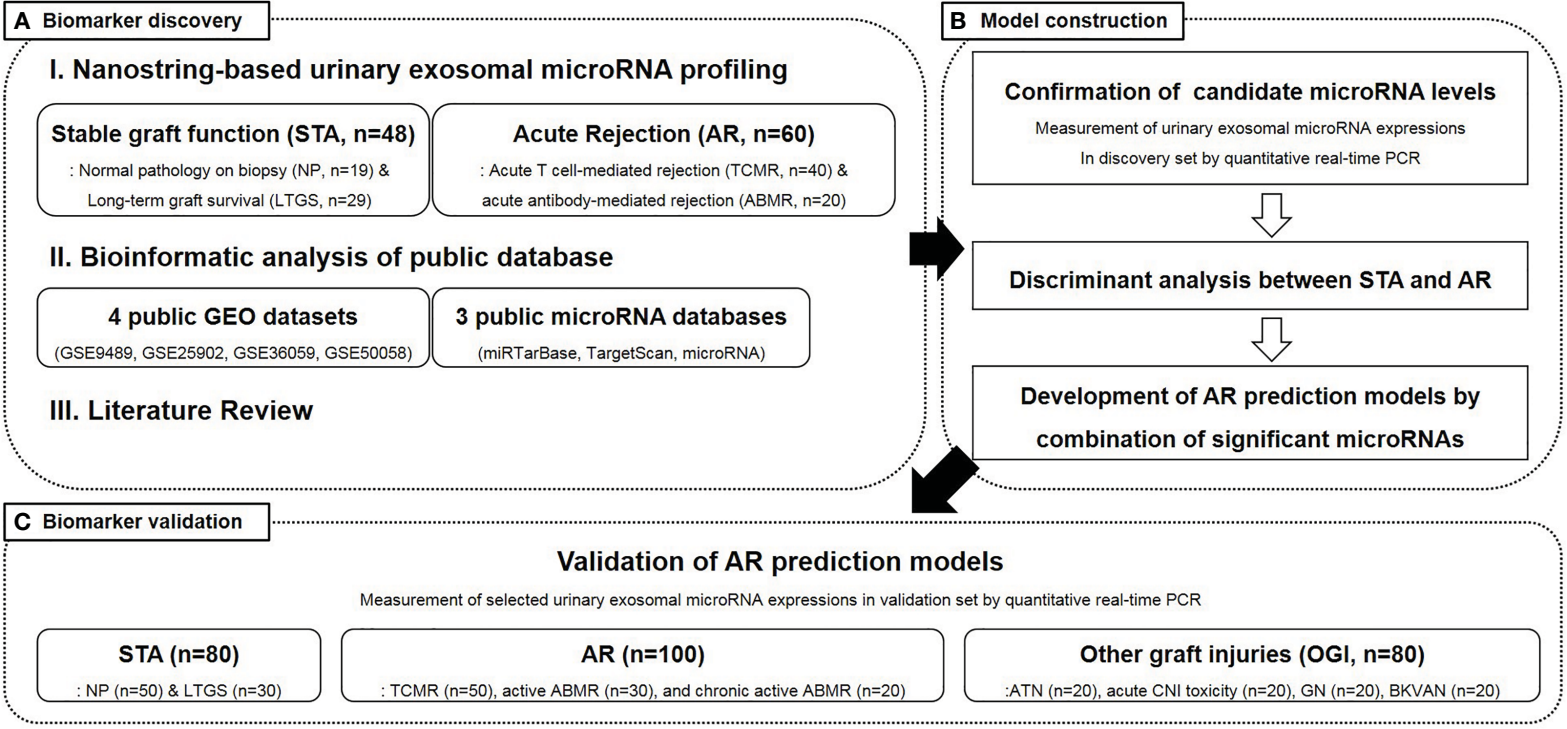
An overview of the study design and participant selection. STA, stable graft function; AR, acute rejection; LTGS, long term graft survival; TCMR, acute T cell-mediated rejection; ABMR, antibody-mediated rejection; GEO, gene expression omnibus; PCR, polymerase chain reaction; ATN, acute tubular necrosis; CNI, calcineurin inhibitor; BKVAN, BK virus-associated nephropathy.

This study was conducted in three sequential stages, as illustrated in **Figure 1**. In the first stage, we aimed to discover candidate microRNAs that could distinguish between kidney transplant recipients with AR and those maintaining stable graft functions. These candidate microRNAs were identified using the following three methods: 1) NanoString-based high-throughput analysis of urinary exosomal microRNAs, 2) meta-analysis of web-based, public microRNA database, and 3) literature reviews. In the second stage, the expression level of each urinary exosomal microRNA candidate was confirmed by quantitative real-time polymerase chain reaction (qPCR) using TaqMan advanced miRNA assays. Subsequently, the microRNAs that could significantly distinguish AR from stable graft function were processed to generate signatures of AR using binary logistic regression. Finally, in the third stage, we tested the diagnostic ability of AR-specific microRNA signatures using urinary samples obtained from independent patient groups.

### 2.2 Study cohort

Study participants were recruited from the Assessment of Immunologic Risk and Tolerance in Kidney Transplantation (ARTKT-1), a multicenter cross-sectional study, which collected transplant-related information as well as blood and urine samples from six different hospitals in Korea. The 368 kidney transplant recipients, who had been enrolled in this study, were divided into a discovery set (n = 108) and a validation set (n = 260) (**Figure 1**). The discovery samples were randomly selected, and there were clearly defined clinical and pathological differences between the AR and stable patients. Out of the 108 recipients in the discovery set, 48 were maintaining stable graft function [STA: normal pathology on protocol biopsy (NP, n = 19) and long-term graft survival (LTGS, n = 29)], while 60 had biopsy-proven AR [acute T cell-mediated rejection (TCMR, n = 40) and active antibody-mediated rejection (aABMR, n = 20)]. Among the 260 recipients in the validation set, 80 were with stable graft function (NP, n = 50 and LTGS, n = 30), 100 with AR [TCMR, n = 50; aABMR, n = 30; and chronic active ABMR (cABMR), n = 20], and the remaining 80 with other graft injuries (OGI). NP applies to recipients with serum creatinine levels < 1.2 mg/dL and without any histological evidence of rejection, calcineurin inhibitor toxicity, acute tubular necrosis, tubulointerstitial inflammation, vascular injury, or glomerulonephritis. LTGS applies to recipients who have maintained serum creatinine levels < 1.2 mg/dL and urinary protein/creatinine ratio < 500 mg/gCr for more than 10 years post-transplantation. Additionally, TCMR and ABMR were diagnosed using graft biopsy, based on the Banff 2017 classification (24, 25), and concurrent elevation of serum creatinine levels, whereas OGI included recipients with acute tubular necrosis (n = 20), acute calcineurin inhibitor toxicity (n = 20), non-specific interstitial fibrosis and tubular atrophy (n = 20), and BK virus-associated nephropathy (n=20).

The age, sex, duration after kidney transplantation, serum creatinine, urinary protein/creatinine ratio, and use of induction and maintenance immunosuppressive drugs, as well as donor type, ABO incompatibility, number of human leukocyte antigen (HLA) mismatches, and other necessary donor information was recorded for each patient at the time of graft biopsy for the biopsy cohort or at the time of visit to the outpatient clinic for the LTGS group. Graft function was assessed based on the estimated glomerular filtration rate (eGFR) using the Chronic Kidney Disease Epidemiology Collaboration (CKD-EPI) formula (26). This study adheres to the Declaration of Istanbul, and the study design was approved by the local institutional review board (#2012-01-030, KHNMC) as well as registered under the Clinical Research Information Service (KCT0001010). Informed consent was obtained from all the patients.

### 2.3 Collection of urine samples and isolation of total RNA from urinary exosomes

Mid-stream, second morning void urine samples were collected, centrifuged at 2,000× *g* for 20 minutes at room temperature, and the supernatant was transferred into RNA later solution (Thermo Scientific, Waltham, MA) and stored at -80°C until further use. Urinary exosomal RNA was isolated from 1 mL of urinary supernatant using spin column-based exoRNeasy serum/plasma midi kits (QIAGEN GmbH, Hilden, Germany), as described previously (11). The quantity (absorbance at 260 nm) and purity (ratio of absorbance at 260 and 280 nm) of RNA were measured using NanoDrop1 ND-2000 UV spectrophotometer (Thermo Scientific).

### 2.4 NanoString-based urinary exosomal microRNA profiling

From the urinary exosome-derived total RNA of each patient’s sample, 80 ng RNA was subjected to high throughput screening for microRNAs using NanoString nCounter system (NanoString, Seattle, WA). Sample processing and microRNA expression analysis were performed according to the manufacturer’s instructions. Each target microRNA was normalized with internal positive controls, and the geometric means of the top 100 expressed targets were calculated using nSolver software in a digital analyzer. Normalized microRNAs were expressed as fold change (FC) values among the groups, and microRNAs fulfilling the criteria of |FC value| ≥ 2 and *p* value ≤ 0.05 in the AR groups, as compared to that in the STA groups were considered as candidates for AR-specific biomarkers.

### 2.5 Analysis of public databases and review of previously published literature to select candidate microRNAs

The potential candidate microRNAs were selected by two approaches. First, Fisher’s exact test was performed to compare between AR-related mRNAs and microRNA target gene sets from three public databases, namely miRTarBase (https://bio.tools/mirtarbase), TargetScan (http://www.targetscan.org/vert_72), and miRbase (http://www.mirbase.org). The mRNAs were selected using GeneMeta R package and categorized in four data sets, according to the approach outlined by Choi et al. (27). Among these, 806 significant mRNAs with a false discovery rate (FDR) < 0.1 and FC > 1.25 were sorted by meta-analysis. The microRNA target gene sets were downloaded from the miRTarBase, TargetScan, and miRBase databases, and 1,110,357 target genes were extracted against 1,802 microRNAs. Among these target genes, only those that appeared in miRBase as well as TargetScan or miRTarBase were finally selected, leading to the extraction of 405,092 genes against 1,760 microRNAs. Subsequently, a gene-set enrichment test was performed on all four datasets. The C3 gene sets (regulatory target gene sets) of the Molecular Signatures Database were used for this analysis, and the enriched microRNAs were sorted by their normalized enrichment scores. Finally, to identify candidate microRNAs, based on literature review, the PubMed database was searched for papers in English, published between 2000 and 2017, using the terms “kidney transplantation,” “acute rejection,” and “microRNA.”

### 2.6 Measurement of microRNA expression using real-time PCR

The candidate microRNAs associated with AR, as detected using NanoString analysis, meta-analysis, and literature review, were subsequently subjected to qPCR. Complementary DNA (cDNA) was synthesized from the extracted RNA using TaqMan advanced miRNA cDNA synthesis kit (Thermo Scientific), according to the manufacturer’s protocol. Initially, each microRNA was subjected to universal 3′ poly-A tailing and 5′ ligation of adaptor sequences, followed by amplification using universal primers to increase the amount of cDNA. This method is advantageous as compared to the traditional reverse transcription PCR with step-loop primers because multiple microRNA targets can be assessed from a single amplified sample.

Commercially available, gene-specific oligonucleotide primers and TaqMan probes were used to measure the levels of each candidate microRNA. The hsa-miR-16-5p was used as an endogenous control for normalization of the microRNAs, and the expression levels were calculated using the comparative cycle threshold (Ct) method (28, 29). Two quality control strategies were implemented. The first-level quality control was based on the levels of hsa-miR-16-5p, and urinary samples exhibiting Ct values ≥ 35 were excluded from this analysis. The second-level quality control was based on the proportion of patients who did not express a particular urinary exosomal microRNA; if a microRNA was absent in ≥ 20% of the enrolled patients, then they were regarded as inadequate candidates for biomarkers.

### 2.7 Statistical analyses

All statistical analyses were performed using SPSS for Windows, version 20.0 (SPSS, Chicago, IL). Baseline clinical data are expressed as mean ± standard deviation (SD) or as the number of patients and corresponding percentages. Independent t-tests, analysis of variance (ANOVA), and χ^2^ tests were performed to compare the baseline characteristics and laboratory observations, as appropriate. Urinary exosomal microRNA expressions were compared among the subgroups using Kruskal–Wallis test, while Dunn’s test was performed for comparisons within each group, since these data are non-normally distributed. Receiver operating characteristic (ROC) curves and areas under the curve (AUC) were constructed to assess the abilities of urinary exosomal microRNAs with respect to prediction of AR in recipients. Differences with *p*-values < 0.05 were considered as statistically significant.

## 3 Results

### 3.1 Clinical characteristics of patients

The baseline demographics and clinical characteristics of the enrolled patients are shown in **Table 1**. In the discovery set, the recipients with AR were younger, predominantly male, had a shorter transplant vintage, lower eGFR, and more severe proteinuria than that of the recipients with STA. Additionally, steroid had been prescribed more frequently in the AR group, as compared to that in the STA group. In the validation set, there was no particular age difference between the patients of STA and AR groups. However, eGFR was lower in patients with AR than in those with STA. Graft function in patients with BK virus-associated nephropathy and OGI was comparable to that in patients with AR. Detailed descriptions of the patients according to each clinicopathological diagnosis as well as their comparisons are presented in **Supplementary Table 1**. In the ABMR group, 75% of the recipients had donor-specific antibodies at the time of graft biopsy, with 45% and 30% having preformed and *de novo* donor specific antibody (DSA), respectively.

**Table 1:** Baseline characteristics and clinical parameters of enrolled patients according to study groups.

	Discovery set (n=108)			Validation set (n=260)			
	STA (n=48)	AR (n=60)	p	STA (n=80)	AR (n=100)	OGI (n=80)	p
Clinicopathologic diagnosis (n, %)	19 NP 29 LTGS	40 TCMR 20 aABMR	–	50 NP 30 LTGS	50 TCMR 30 aABMR 20 cABMR	20 ATN 20 GN 20 IFTA 20 BKVAN	–
Age (years)	53.4 ± 10.7	47.6 ± 11.1	0.007	47.6 ± 12.1	47.4 ± 11.4	47.4 ± 13.1	0.995
Sex (Male, %)	19 (39.6)	39 (65.0)	0.008	42 (52.5)	69 (69.0)	60 (75.0)	0.008
Deceased donor KT (n, %)	13 (27.1)	27 (45.0)	0.055	20 (25.0)	31 (38.8)	35 (43.8)	0.036
Duration after KT (year)	9.9 ± 8.9	1.5 ± 2.6	<0.001	5.8 ± 7.4	4.4 ± 4.4	2.7 ± 4.5	0.003
ABO incompatible KT (n, %)	2 (4.2)	14 (23.3)	0.006	5 (6.2)	8 (8.0)	5 (6.2)	0.864
HLA mismatching (n)	2.6 ± 1.6	3.7 ± 1.5	0.001	3.0 ± 1.6	3.2 ± 1.6	3.4 ± 1.8	0.291
eGFR (ml/min per 1.73 m^2^)	69.7 ± 12.8	37.6 ± 22.0	<0.001	77.2 ± 20.6	33.4 ± 15.4	39.4 ± 17.0	<0.001
Urine PCR (mg/gCr)	175 ± 293	1050 ± 1551	<0.001	232 ± 380	1574 ± 1974	1245 ± 1950	<0.001
Induction immunosuppression			0.021				0.320
Basiliximab (n, %)	40 (83.3)	38 (63.3)		65 (81.2)	87 (87.0)	63 (78.8)	
Anti-thymocyte globulin (n, %)	8 (16.7)	22 (36.7)		15 (18.8)	13 (13.0)	17 (21.2)	
Maintenance immunosuppression							
Steroid (n, %)	30 (62.5)	52 (86.7)	0.004	70 (87.5)	89 (89.0)	74 (92.5)	0.565
Calcineurin inhibitor (n, %)	44 (91.7)	57 (95.0)	0.698	77 (96.2)	92 (92.0)	77 (96.2)	0.336
Mycophenolate mofetil (n, %)	31 (64.6)	45 (75.0)	0.239	71 (88.8)	84 (84.0)	64 (80.0)	0.315
mTOR inhibitor (n, %)	5 (10.4)	4 (6.7)	0.507	5 (6.2)	7 (7.0)	5 (6.2)	0.972
Donor age (years)	33.9 ± 15.3	46.1 ± 12.7	<0.001	41.3 ± 14.7	45.5 ± 17.6	49.3 ± 12.5	0.006
Donor sex (Male, %)	30 (62.5)	35 (58.3)	0.660	44 (55.0)	48 (48.0)	38 (47.5)	0.633

Data are expressed as mean ± standard deviation or number (percentage).

STA, stable graft function; AR, acute rejection; cABMR, chronic active antibody-mediated rejection; OGI, other graft injuries; NP, normal pathology; LTGS, long term graft survival; TCMR, acute T cell-mediated rejection; aABMR, active antibody-mediated rejection; cABMR, chronic active antibody-mediated rejection; ATN, acute tubular necrosis; GN, glomerulonephritis; IFTA, interstitial fibrosis and tubular atrophy; BKVAN, BK virus associated nephropathy; KT, kidney transplantation; HLA, human leukocyte antigen; eGFR, estimated glomerular filtration rate; PCR, protein-to-creatinine ratio; mTOR, mammalian target of rapamycin.

### 3.2 Selection of AR-specific microRNA candidates using NanoString transcriptomics

To screen and identify microRNA candidates using a transcriptome-based approach, we isolated urinary exosomal RNAs from urine samples of patients in the discovery set. The median quantity of total RNA was 0.342 μg (25 and 75 percentiles; 0.295 and 0.466 μg, respectively), and the median purity of total RNA was 1.485 (25 and 75 percentiles; 1.340 and 1.610, respectively). All samples qualified in the quality control tests and were processed for NanoString analysis. Of the 798 microRNAs that were assessed, 14 fulfilled the selection criteria and were selected as candidate microRNAs for predicting AR (**Supplementary Figure 1**). Notably, 1 microRNA (hsa-miR-197-5p) had a significantly higher expression in the AR group, as compared to that in the STA group, whereas the remaining 13 microRNA expressions were significantly decreased in the patients with AR.

### 3.3 Selection of AR-specific microRNA candidates using bioinformatics and literature review

Fisher’s analytical test revealed five microRNAs with the lowest FDR, while gene enrichment analysis discovered five additional microRNAs with the lowest normalized enrichment score and a filtered FDR < 0.1. Furthermore, literature review led to the discovery of five microRNAs that were differentially expressed in patients with AR (21, 30).

Therefore, we identified 29 microRNAs (14 from NanoString analysis, 10 from bioinformatics analysis, and 5 from literature review) as candidate biomarkers of AR (**Table 2**).

**Table 2:** List of AR-specific microRNA candidates selected from Nanostring, bioinformatics and literature review.

Nanostring analysis	Bioinformatic analysis	Literature review
hsa-miR-197-5p	hsa-miR-21-5p	hsa-miR-142-3p
hsa-miR-575	hsa-miR-30a-3p	hsa-miR-142-5p
hsa-miR-489-3p	hsa-miR-124-3p	hsa-miR-155-5p
hsa-miR-4532	hsa-miR-146a-5p	hsa-miR-223-3p
hsa-miR-4516	hsa-miR-335-5p	hsa-miR-210-3p
hsa-miR-4488	hsa-miR-31-5p	
hsa-miR-320e	hsa-miR-186-5p	
hsa-miR-3195	hsa-miR-324-3p	
hsa-miR-3185	hsa-miR-373-3p	
hsa-miR-3158-3p	hsa-miR-518a-5p	
hsa-miR-1915-3p		
hsa-miR-187-3p		
hsa-miR-1305		
hsa-miR-1268a		

### 3.4 Expression of candidate microRNAs in urinary exosomes

**Figure 2:**
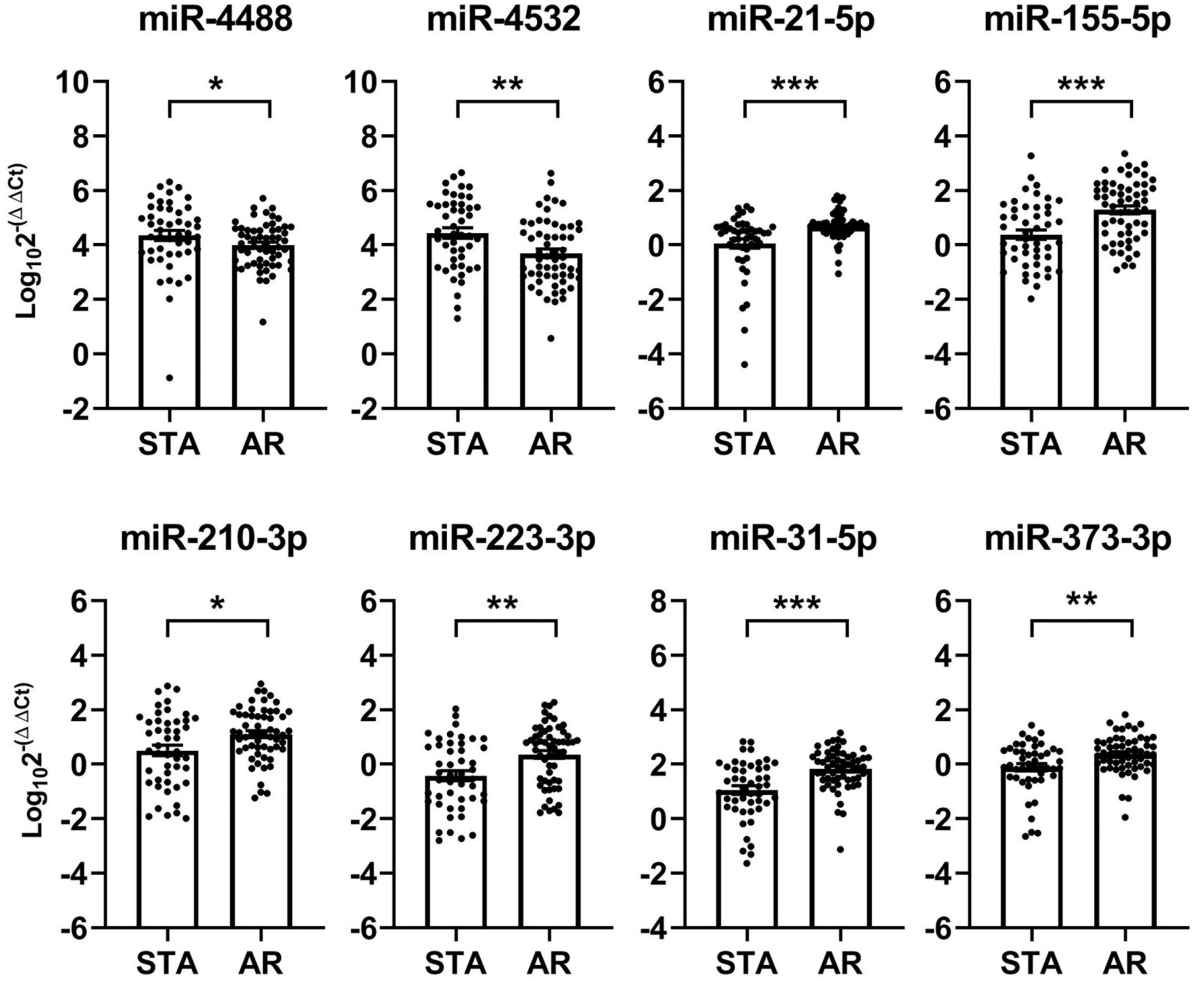
Identification of acute rejection-specific urinary exosomal microRNAs in discovery set. Quantitative real-time polymerase chain reaction (qPCR) analysis of the 29 candidate microRNAs selected from NanoString analysis, bioinformatics analysis, and literature review reveals that 8 (as shown below) are differentially expressed in patients with acute rejection, as compared to their corresponding expressions in patients maintaining stable graft functions. Each microRNA level has been normalized using hsa-miR-16-5p and expressed as delta delta cycle threshold (ddCt) value after log transformation. STA, stable graft function; AR, acute rejection. ^*^p < 0.05, ^**^p < 0.01, ^***^p < 0.001.

The expression levels of 3 of the 29 microRNAs, namely hsa-miR-1268a, hsa-miR-1305, and hsa-miR-319, could not be measured due to the commercial unavailability of their primers. Therefore, we assessed the expression levels of the remaining 26 microRNAs along with hsa-miR-16-5p (a reference microRNA) in the urinary exosomal fractions of the enrolled patients. qPCR analysis revealed that eight microRNAs exhibited significant differences between their urinary exosomal expressions in the AR and STA groups (**Figure 2**). In particular, two microRNAs, hsa-miR-4488 and hsa-miR-4532, were downregulated, while six microRNAs, hsa-miR-21-5p, hsa-miR-155-5p, hsa-miR-210-3p, hsa-miR-223-3p, hsa-miR-31-5p, and hsa-miR-373-3p, are upregulated in the AR group, as compared to that in the STA group. On the contrary, 14 microRNAs failed to qualify the quality control assessment (detection rate of ≤ 80%), and 4 microRNAs did not exhibit any between-group differences in their corresponding expression levels. Subgroup analysis revealed that the levels of two microRNAs, hsa-miR-21-5p and hsa-miR-210-3p were higher in the NP group than in the LTGS group, while no significant differences were observed between the TCMR and ABMR groups (**Supplementary Figure 2**). The levels of the selected urinary exosomal microRNAs were not significantly associated with the presence or type of DSA (**Supplementary Figure 3**).

### 3.5 Generation of AR-specific microRNA signature

**Figure 3:**
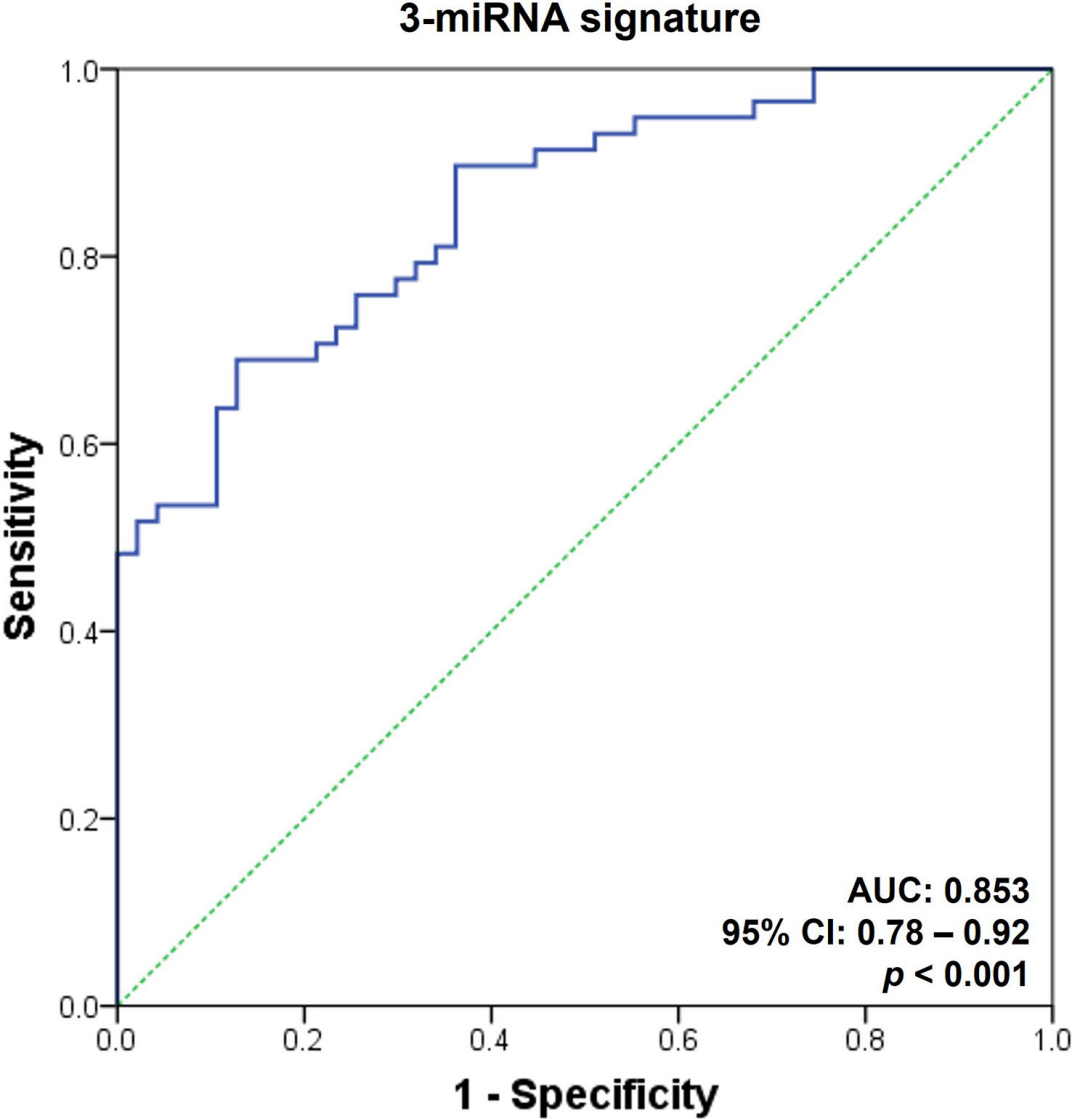
Receiver operating characteristic curves for the diagnosis of acute rejection in discovery set. An acute rejection (AR) prediction model was generated using a combination of AR-specific urinary exosomal microRNAs. The receiver operating characteristic (ROC) curves of the 3-microRNA signature (hsa-miR-21-5p, hsa-miR-31-5p, and hsa-miR-4532) is presented. AUC, area under the curve; CI, confidence interval; PPV, positive predictive value; NPV, negative predictive value.

The ROC curves were generated to evaluate the diagnostic ability of the selected microRNAs as AR biomarkers (**Figure 3**). Since a single microRNA was not sufficient for the diagnosis of AR (AUC ranging from 0.63 to 0.73), we integrated the urinary exosomal microRNAs and generated AR-specific signatures. Using a forward stepwise logistic regression method, we determined the minimal microRNA dataset with the best performance regarding AR detection. Incidentally, this was a set of 3-microRNA signatures, composed of hsa-miR-4532, hsa-miR-21-5p, and hsa-miR-31-5p (AUC = 0.85, 95% confidence interval [CI] = 0.78 - 0.92, *p* < 0.001; **Figure 3**).

### 3.6 Validation of AR-specific urinary exosomal microRNA signature in an independent cohort

**Figure 4:**
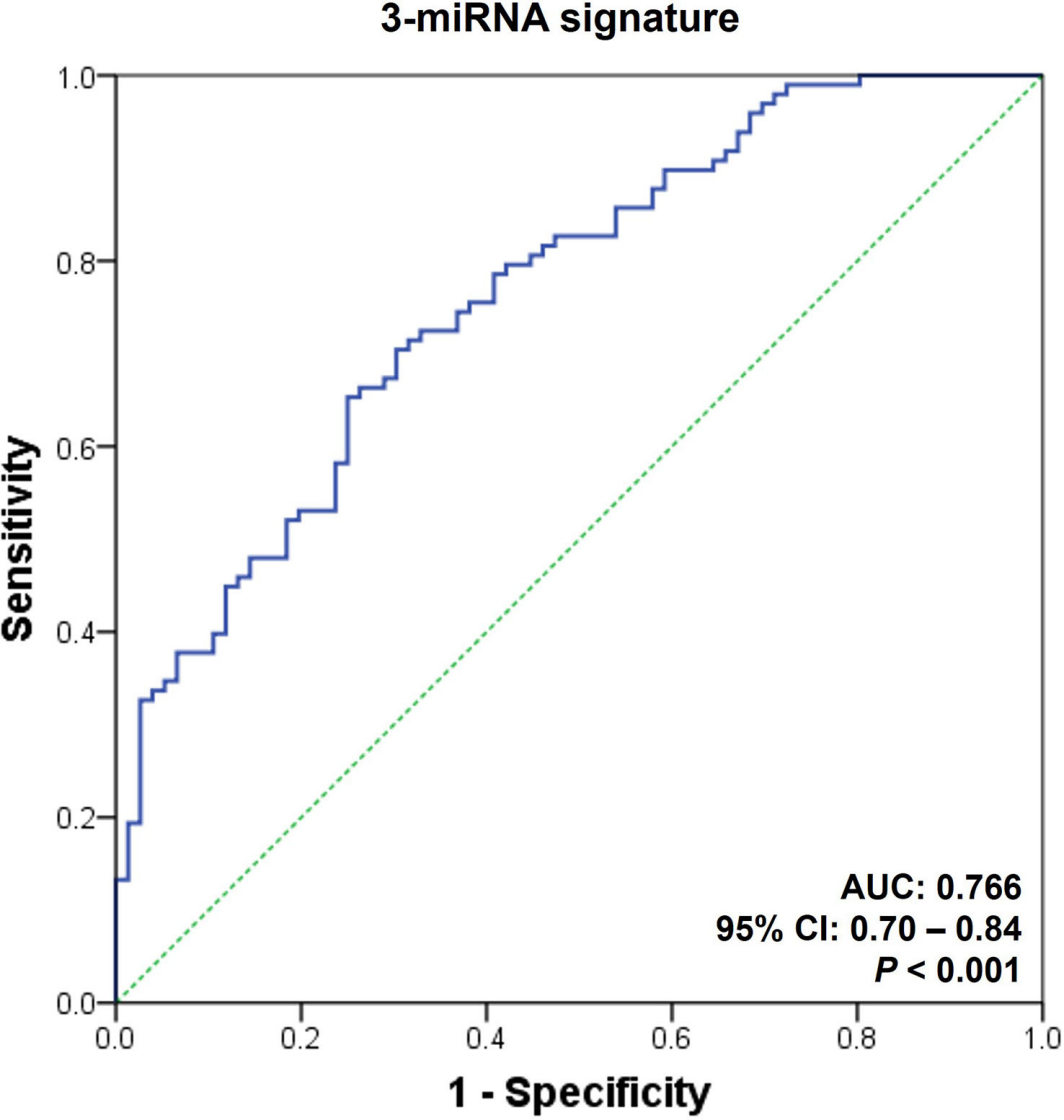
Receiver operating characteristic curves for the diagnosis of acute rejection in validation set. An acute rejection (AR) prediction signature was generated by analyzing the discovery set; subsequently, it was applied to an independent validation cohort. The receiver operating characteristic (ROC) curves of the 3-microRNA signature (hsa-miR-21-5p, hsa-miR-31-5p, and hsa-miR-4532) is presented. AUC, area under the curve; CI, confidence interval; PPV, positive predictive value; NPV, negative predictive value.

Finally, we measured the expression levels of the selected microRNAs in the urinary exosomal fractions of independent recipients (n = 260) to validate the performance of the AR-specific microRNA signature. The fixed cut-off value set in the discovery cohort was applied in the assessment process of the validation cohort. The expressions of the eight microRNAs are displayed in **Supplementary Figure 4**. We discovered that the 3-microRNA signature was fairly successful in distinguishing AR from biopsy-proven rejection, as well as other conditions, including BK virus-associated nephropathy, acute tubular necrosis, or calcineurin inhibitor toxicity (AUC = 0.77, 95% CI = 0.70 - 0.84, *p* < 0.001; **Figure 4** and **Table 3**).

**Table 3:** Prediction performance of the microRNA signature in the validation set.

	AUC (95% CI)	Sensitivity (95% CI)	Specificity (95% CI)	PPV (95% CI)	NPV (95% CI)	Accuracy (95% CI)	Cut-off point	p value
Three-microRNA signature	0.77 (0.70 to 0.84)	0.99 (0.95 to 1.00)	0.26 (0.17 to 0.38)	0.63 (0.60 to 0.67)	0.95 (0.73 to 0.99)	0.67 (0.60 to 0.74)	0.65	< 0.001

AUC, area under the curve; CI, confidence interval; PPV, positive predictive value; NPV, negative predictive value.

## 4 Discussion

In the present study, we extensively investigated the urinary exosomal microRNA profiles of kidney transplant recipients and attempted to determine their clinical utility as non-invasive biomarkers of AR. We identified differentially expressed urinary exosomal microRNAs in recipients with AR using NanoString-based transcriptomics, public databases, as well as previously published literature and successfully validated a urinary exosomal microRNA signature that could effectively discriminate AR from STA as well as OGIs, including BK virus-associated nephropathy, acute tubular necrosis, or calcineurin inhibitor toxicity. Altogether, these data suggest that urinary exosomal microRNAs may be a useful source of novel, non-invasive biomarkers for renal allograft rejection.

Renal allograft biopsy is currently considered the gold standard for diagnosing acute rejection in kidney transplant recipients. However, graft biopsy possesses several complications such as bleeding, infection, and fistula formation. However, this procedure can lead to complications such as bleeding, infection, and fistula formation. Additionally, since biopsy tissue reveals only a small portion of the kidney, it can hinder the accurate diagnosis of transplant pathology. A recent study compared pathologic scores of whole kidney tissues and biopsy specimens that were obtained from the same kidneys. The examinations of biopsy specimens consistently resulted in lower pathologic scores, thus underestimating the severity of the entire kidney tissues, particularly in the case of tubulointerstitial fibrosis (31). To overcome the limitations associated with biopsy, the identification of reliable biomarkers that accurately represent the pathologic process of the whole kidneys is considered a viable solution. Additionally, the implementation of digital pathology with the assistance of artificial intelligence is a potential future direction to reduce sampling bias and achieve accurate interpretation of pathologic findings (32, 33).

Previous studies have consistently demonstrated that microRNAs have the potential to function as biomarkers of transplant pathologies such as acute and chronic rejection (21, 30, 34–37), delayed graft function (37–41), and tubulointerstitial fibrosis (20, 22, 42, 43). Furthermore, significant changes in microRNA profiles have been observed in cases of subclinical rejection, suggesting that these alterations may occur before elevations in serum creatinine levels and thus can be utilized in monitoring kidney transplant recipients while decreasing the need for surveillance biopsies (21).

Urinary exosomes have several unique advantages over urinary cell pellets or supernatant as a source of biomarkers. These urinary exosomes reflect the local pathophysiological status of renal allografts, since they are mostly derived from renal epithelial cells (6, 44, 45). The circulating exosomes are unlikely to leak into the urinary space from blood due to their relatively large sizes, as compared to that of the glomerular pores (30 - 100 nm diameter of exosomes vs. 4 nm diameter of slit diaphragm) (46). Moreover, urinary exosomes are surrounded by a lipid bilayer membrane, which protects intra-exosomal RNAs from degradation by urinary ribonucleases (47). A direct comparison among urinary exosomes, supernatant, and cell pellets revealed a superior yield of microRNAs from exosomes, as compared to the yield from the other two (47, 48). Furthermore, alterations in urinary exosomal microRNA profiles are well correlated with clinical activity of patients with lupus nephritis, whereas the changes in the urinary supernatant or cell pellet microRNAs are not (15). Therefore, we concluded that urinary exosomes would be a better source of microRNA biomarkers than the urinary supernatants or cell pellets.

In a previous study, we had demonstrated that the spin column-based enrichment of urinary exosomes can substitute the traditional ultracentrifugation-based method, which is labor-intensive and difficult to implement in clinical applications (11). However, the limited concentration of the urinary exosomal RNAs is a major concern during the selection of high-throughput platforms for the measurement of the microRNAs. The amount of total exosomal RNAs extracted from 1mL of urine is as low as 80 ng, thereby indicating that microarray or RNA-sequencing (RNA-seq) may result in inadequate data acquisition. Although recent advances in sequencing techniques have demonstrated the possibility of analyzing whole transcriptomic data using as little as 1 ng of total RNA, further research is necessary to confirm the sensitivity and reproducibility of this novel method (49). Several studies have previously demonstrated that NanoString can be utilized for high-throughput analysis of urinary exosomal microRNAs using only 100 ng of total RNA, which is in accordance with the prerequisite for this study (50–53). Therefore, we speculated that NanoString would be a better platform for quantifying microRNAs in this experiment, as compared to the performances of microarray analysis or RNA-seq. With only 80 ng of total RNA for the assessment of microRNAs, we obtained results that were adequate for their profile analyses.

Majority of the studies that have investigated biomarkers for rejection with respect to kidney transplantation have used different threshold values for the discovery and validation stages, thereby limiting their application in clinical practice (54). In fact, we have recently demonstrated that fixed cut-off values can be useful in urinary mRNA-based non-invasive diagnosis of AR (55). Hence, an important consideration of our study is the application of a fixed cut-off value during the assessment of the validation cohort. Our microRNA signature maintains a comparable diagnostic ability using fixed cut-off value in an independent validation cohort, suggesting that the signature is highly likely to be reproducible in clinical practice.

The differentially expressed urinary exosomal microRNAs have various pathophysiological characteristics (**Table 4**). The hsa-miR-21-5p is consistently upregulated in various clinical and experimental kidney diseases, including transplantation (65–70), although its exact contributory mechanisms are still under investigation. In experimental renal ischemia-reperfusion injury models, elevated hsa-miR-21-5p levels is associated with deteriorations in renal function and increased the expressions of tumor necrosis factor-α as well as interleukin-6 in renal tubular epithelial cells, thereby suggesting that it has a pro-inflammatory role in acute kidney damage (56). In addition, miR-21-5p promotes inflammation by activating the SPRY1/ERK/NF-kB signal pathways in unilateral ureteral obstruction mice (57, 58). Moreover, hsa-miR-21-5p is involved in the polarization of T lymphocytes towards T helper 17 cells in patients with IgA nephropathy (59). In accordance with these reports, we observed that patients with AR exhibit increased levels of hsa-miR-21-5p in urinary exosomes, thus indicating enhanced inflammation in renal allografts. On the contrary, the pathophysiological role of hsa-miR-31a-5p in kidney diseases has not yet been investigated extensively. Reportedly, hsa-miR-31a-5p plays a role in the generation of oxidative stress, and it is upregulated in the urinary exosomes of patients with diabetic nephropathy (45, 60). In contrast, another study showed that miR-31-5p was found to be declined in patients with diabetic nephropathy and had a suppressive effect on the apoptosis, inflammation, and oxidative stress in podocytes under high glucose condition (61). The urinary hsa-miR-4532 expression is significantly lower in patients with biopsy-proven diabetic kidney disease than that in healthy controls (71). Moreover, its levels are negatively associated with the degree of tubulointerstitial inflammation and long-term renal function decline, thereby suggesting that hsa-miR-4532 may be a biomarker of intra-renal inflammation in kidney diseases (72). On the other hand, high levels of macrophage-derived exosomal miR-4532 activated NF-kB and promotes endothelial cell injury through inhibiting Sphingosine-1-phosphate expression (62). Dysregulated expression of circulating hsa-miR-4532 has also been documented in various pathological conditions, such as malignancy, endometriosis, and cerebral infarction (63, 64, 73, 74).

**Table 4:** Biological functions of acute rejection-specific microRNAs.

	Descriptions	Reference
hsa-miR-21-5p	Upregulation of mitogen-activated protein kinase kinase 3 (MKK3)	(56)
	Activation of SPRY1/ERK/NF-kB pathway	(57)
	Inhibition of UUO-induced kidney fibrosis	(58)
	Th17 cell differentiation	(59)
hsa-miR-31-5p	Promotion of oxidative stress and vascular smooth muscle cell migration	(60)
	Amelioration of high-glucose induced podocytes injury	(61)
hsa-miR-4532	Upregulation of NF-kB through sphingosine-1-phosphate	(62)
	Activation of LDOC1-dependent STAT3 signaling pathway	(63)
	Downregulation of hypermethylated in cancer-1 (*HIC-1*) gene	(64)

SPRY1, Sprouty RTK Signaling Antagonist 1; ERK, extracellular signal-regulated kinase; NF-kB, nuclear factor kappa-light-chain-enhancer of activated B cells; UUO, unilateral ureteral obstruction; LDOC1, leucine zipper, downregulated in cancer 1; STAT3, Signal transducer and activator of transcription 3.

This study has certain limitations. We observed that the discriminative power of the AR prediction models developed in the discovery set decreased slightly during the assessment of the validation cohort (**Figures 3**, **4**). One probable cause for this discrepancy might be the differences in the baseline clinical parameters of the patients included in the discovery and validation sets (**Table 2**). The inclusion of patients with BK virus-associated nephropathy and OGIs exclusively in the validation stage might also have negative impacts on the performance of the AR prediction model since this model was generated based on comparisons between the STA and AR groups. Nonetheless, the difference was small and statistically insignificant (AUC = 0.77 and 95% CI = 0.70 – 0.84 vs. AUC = 0.85 and 95% CI = 0.78 – 0.92; **Figures 3**, **4**). Furthermore, the primary purpose of this study was to generate microRNA signatures capable of distinguishing AR from STA as well as other causes of acute kidney injury, such as BK virus-associated nephropathy or acute tubular necrosis, all of which are important diagnostic categories that need to be differentiated in clinical practice. Therefore, the fair discriminative power of our microRNA signature indicates the generalizability of this model for the assessment of kidney transplant recipients. Another issue is the inadequate amplification of approximately half of the microRNAs (14/29, 48.3%), which were selected during the discovery process, by qPCR in > 20% of the urinary samples, thereby causing them to be rejected as biomarkers of AR. One possible explanation is the use of TaqMan advanced miRNA assays that employ a universal reverse transcription step instead of a target-specific reverse transcription step during cDNA synthesis from microRNAs. Hence, in spite of reducing the time and labor required to synthesize cDNA for qPCR with multiple microRNAs, the specificity of these assays is reportedly low, as compared to that of qPCR using standard, sequence-specific, stem-loop primers. Nevertheless, we proceeded to the next step without re-assessing these microRNAs since eight microRNAs had been detected as differentially expressed in the AR group, and that was sufficient for the development of AR prediction signatures. Finally, we combined recipients with NP and LTGS as a stable group in the discovery set, despite the significant difference in transplant vintages, to identify AR-specific microRNA candidates. The differences in post-transplantation duration might affect microRNA expression profiles, as shown in **Supplementary Figure 2**. Nonetheless, the current study focused on discovering AR-specific microRNA signatures that can be applied irrespective of transplant vintage. Previous investigations on AR biomarkers has also shown significant variability in the duration post-transplantation, ranging from 2 months to 8 years (8, 75). Further investigations are needed to investigate the temporal changes in microRNA profiles in stable kidney transplant recipients.

In conclusion, this study demonstrated that urinary exosomal microRNA profiles could serve as potential biomarkers for the non-invasive diagnosis of AR in kidney transplant recipients. We expect that future prospective trials will clarify the clinical relevance of our AR-prediction signature.

## Data availability statement

The datasets presented in this study can be found in online repositories. The names of the repository/repositories and accession number(s) can be found below: GSE230858 (GEO).

## Ethics statement

The studies involving human participants were reviewed and approved by #2012-01-030, KHNMC. The patients/participants provided their written informed consent to participate in this study.

## Author contributions

J-WS: formal analysis, methodology, software, and writing original draft. YL: funding acquisition, formal analysis, methodology, software, and writing original draft. DT: formal analysis. YK, J-YM, SJ, JK, HH, K-HJ, HJ, S-YL: supervision. BC, C-DK, JP, YHK: sample and data collection, supervision. JS: methodology, software, and formal analysis. S-HL: funding acquisition, conceptualization, methodology, formal analysis, and writing original draft. All authors contributed to the article and approved the submitted version.

## References

[1] Hariharan S Israni AK Danovitch G. Long-term survival after kidney transplantation. N Engl J Med (2021) 385:729–43. doi: 10.1056/NEJMra2014530 34407344

[2] Menon MC Murphy B Heeger PS. Moving biomarkers toward clinical implementation in kidney transplantation. J Am Soc Nephrol (2017) 28:735–47. doi: 10.1681/ASN.2016080858 PMC5328171 28062570

[3] Caruso S Poon IKH. Apoptotic cell-derived extracellular vesicles: more than just debris. Front Immunol (2018) 9:1486. doi: 10.3389/fimmu.2018.01486 30002658 PMC6031707

[4] ELA S Mager I Breakefield XO Wood MJ. Extracellular vesicles: biology and emerging therapeutic opportunities. Nat Rev Drug Discovery (2013) 12:347–57. doi: 10.1038/nrd3978 23584393

[5] Weber JA Baxter DH Zhang S Huang DY Huang KH Lee MJ. The microRNA spectrum in 12 body fluids. Clin Chem (2010) 56:1733–41. doi: 10.1373/clinchem.2010.147405 PMC4846276 20847327

[6] Miranda KC Bond DT McKee M Skog J Paunescu TG Da Silva N. Nucleic acids within urinary exosomes/microvesicles are potential biomarkers for renal disease. Kidney Int (2010) 78:191–9. doi: 10.1038/ki.2010.106 PMC4451567 20428099

[7] Karpman D Stahl AL Arvidsson I. Extracellular vesicles in renal disease. Nat Rev Nephrol (2017) 13:545–62. doi: 10.1038/nrneph.2017.98 28736435

[8] El Fekih R Hurley J Tadigotla V Alghamdi A Srivastava A Coticchia C. Discovery and validation of a urinary exosome mRNA signature for the diagnosis of human kidney transplant rejection. J Am Soc Nephrol (2021) 32:994–1004. doi: 10.1681/ASN.2020060850 33658284 PMC8017553

[9] Lim JH Lee CH Kim KY Jung HY Choi JY Cho JH. Novel urinary exosomal biomarkers of acute T cell-mediated rejection in kidney transplant recipients: a cross-sectional study. PLoS One (2018) 13:e0204204. doi: 10.1371/journal.pone.0204204 30226858 PMC6143249

[10] Lv LL Feng Y Wen Y Wu WJ Ni HF Li ZL. Exosomal CCL2 from tubular epithelial cells is critical for albumin-induced tubulointerstitial inflammation. J Am Soc Nephrol (2018) 29:919–35. doi: 10.1681/ASN.2017050523 PMC5827595 29295871

[11] Kim MH Lee YH Seo JW Moon H Kim JS Kim YG. Urinary exosomal viral microRNA as a marker of BK virus nephropathy in kidney transplant recipients. PloS One (2017) 12:e0190068. doi: 10.1371/journal.pone.0190068 29267352 PMC5739476

[12] Xie Y Jia Y Cuihua X Hu F Xue M Xue Y. Urinary exosomal MicroRNA profiling in incipient type 2 diabetic kidney disease. J Diabetes Res (2017) 2017:6978984. doi: 10.1155/2017/6978984 29038788 PMC5605810

[13] Min QH Chen XM Zou YQ Zhang J Li J Wang Y. Differential expression of urinary exosomal microRNAs in IgA nephropathy. J Clin Lab Anal (2018) 32:e22226. doi: 10.1002/jcla.22226 28383146 PMC6816951

[14] Huang Z Zhang Y Zhou J Zhang Y. Urinary exosomal miR-193a can be a potential biomarker for the diagnosis of primary focal segmental glomerulosclerosis in children. BioMed Res Int (2017) 2017:7298160. doi: 10.1155/2017/7298160 28246603 PMC5303601

[15] Perez-Hernandez J Forner MJ Pinto C Chaves FJ Cortes R Redon J. Increased urinary exosomal MicroRNAs in patients with systemic lupus erythematosus. PLoS One (2015) 10:e0138618. doi: 10.1371/journal.pone.0138618 26390437 PMC4577109

[16] Sigdel TK Ng YW Lee S Nicora CD Qian WJ Smith RD. Perturbations in the urinary exosome in transplant rejection. Front Med (Lausanne) (2014) 1:57. doi: 10.3389/fmed.2014.00057 25593928 PMC4292055

[17] Cheng L Sun X Scicluna BJ Coleman BM Hill AF. Characterization and deep sequencing analysis of exosomal and non-exosomal miRNA in human urine. Kidney Int (2014) 86:433–44. doi: 10.1038/ki.2013.502 24352158

[18] Thongboonkerd V. Roles for exosome in various kidney diseases and disorders. Front Pharmacol (2019) 10:1655. doi: 10.3389/fphar.2019.01655 32082158 PMC7005210

[19] Jing H Tang S Lin S Liao M Chen H Zhou J. The role of extracellular vesicles in renal fibrosis. Cell Death Dis (2019) 10:367. doi: 10.1038/s41419-019-1605-2 31068572 PMC6506498

[20] Maluf DG Dumur CI Suh JL Scian MJ King AL Cathro H. The urine microRNA profile may help monitor post-transplant renal graft function. Kidney Int (2014) 85:439–49. doi: 10.1038/ki.2013.338 PMC3946645 24025639

[21] Lorenzen JM Volkmann I Fiedler J Schmidt M Scheffner I Haller H. Urinary miR-210 as a mediator of acute T-cell mediated rejection in renal allograft recipients. Am J Transplant (2011) 11:2221–7. doi: 10.1111/j.1600-6143.2011.03679.x 21812927

[22] Scian MJ Maluf DG David KG Archer KJ Suh JL Wolen AR. MicroRNA profiles in allograft tissues and paired urines associate with chronic allograft dysfunction with IF/TA. Am J Transplant (2011) 11:2110–22. doi: 10.1111/j.1600-6143.2011.03666.x PMC3184368 21794090

[23] Jung SW Cho WH Seo JW Kim YG Moon JY Kim JS. Urine exosomal bkv-miR-B1-5p and BK virus nephropathy in kidney transplant recipients. J Infect Dis (2022). doi: 10.1093/infdis/jiac440 PMC10175067 36374933

[24] Jeong HJ. Diagnosis of renal transplant rejection: banff classification and beyond. Kidney Res Clin Pract (2020) 39:17–31. doi: 10.23876/j.krcp.20.003 32164120 PMC7105630

[25] Haas M Loupy A Lefaucheur C Roufosse C Glotz D Seron D. The banff 2017 kidney meeting report: revised diagnostic criteria for chronic active T cell-mediated rejection, antibody-mediated rejection, and prospects for integrative endpoints for next-generation clinical trials. Am J Transplant (2018) 18:293–307. doi: 10.1111/ajt.14625 29243394 PMC5817248

[26] Levey AS Stevens LA Schmid CH Zhang YL Castro AF 3rd Feldman HI. A new equation to estimate glomerular filtration rate. Ann Intern Med (2009) 150:604–12. doi: 10.7326/0003-4819-150-9-200905050-00006 PMC2763564 19414839

[27] Choi JK Yu U Kim S Yoo OJ. Combining multiple microarray studies and modeling interstudy variation. Bioinformatics (2003) 19 Suppl 1:i84–90. doi: 10.1093/bioinformatics/btg1010 12855442

[28] Lange T Stracke S Rettig R Lendeckel U Kuhn J Schluter R. Identification of miR-16 as an endogenous reference gene for the normalization of urinary exosomal miRNA expression data from CKD patients. PLoS One (2017) 12:e0183435. doi: 10.1371/journal.pone.0183435 28859135 PMC5578666

[29] Schwarzenbach H da Silva AM Calin G Pantel K. Data normalization strategies for MicroRNA quantification. Clin Chem (2015) 61:1333–42. doi: 10.1373/clinchem.2015.239459 PMC4890630 26408530

[30] Anglicheau D Sharma VK Ding R Hummel A Snopkowski C Dadhania D. MicroRNA expression profiles predictive of human renal allograft status. Proc Natl Acad Sci U S A (2009) 106:5330–5. doi: 10.1073/pnas.0813121106 PMC2663998 19289845

[31] Girolami I Gambaro G Ghimenton C Beccari S Calio A Brunelli M. Pre-implantation kidney biopsy: value of the expertise in determining histological score and comparison with the whole organ on a series of discarded kidneys. J Nephrol (2020) 33:167–76. doi: 10.1007/s40620-019-00638-7 31471818

[32] Girolami I Pantanowitz L Marletta S Hermsen M van der Laak J Munari E. Artificial intelligence applications for pre-implantation kidney biopsy pathology practice: a systematic review. J Nephrol (2022) 35:1801–8. doi: 10.1007/s40620-022-01327-8 PMC9458558 35441256

[33] Eccher A Neil D Ciangherotti A Cima L Boschiero L Martignoni G. Digital reporting of whole-slide images is safe and suitable for assessing organ quality in preimplantation renal biopsies. Hum Pathol (2016) 47:115–20. doi: 10.1016/j.humpath.2015.09.012 26547252

[34] Matz M Fabritius K Lorkowski C Durr M Gaedeke J Durek P. Identification of T cell-mediated vascular rejection after kidney transplantation by the combined measurement of 5 specific MicroRNAs in blood. Transplantation (2016) 100:898–907. doi: 10.1097/TP.0000000000000873 26444957

[35] Vitalone MJ Sigdel TK Salomonis N Sarwal RD Hsieh SC Sarwal MM. Transcriptional perturbations in graft rejection. Transplantation (2015) 99:1882–93. doi: 10.1097/TP.0000000000000809 PMC5221567 26154388

[36] Rascio F Pontrelli P Accetturo M Oranger A Gigante M Castellano G. A type I interferon signature characterizes chronic antibody-mediated rejection in kidney transplantation. J Pathol (2015) 237:72–84. doi: 10.1002/path.4553 25925804

[37] Wilflingseder J Regele H Perco P Kainz A Soleiman A Muhlbacher F. miRNA profiling discriminates types of rejection and injury in human renal allografts. Transplantation (2013) 95:835–41. doi: 10.1097/TP.0b013e318280b385 PMC3677100 23511211

[38] Khalid U Newbury LJ Simpson K Jenkins RH Bowen T Bates L. A urinary microRNA panel that is an early predictive biomarker of delayed graft function following kidney transplantation. Sci Rep (2019) 9:3584. doi: 10.1038/s41598-019-38642-3 30837502 PMC6401030

[39] Amrouche L Desbuissons G Rabant M Sauvaget V Nguyen C Benon A. MicroRNA-146a in human and experimental ischemic AKI: CXCL8-dependent mechanism of action. J Am Soc Nephrol (2017) 28:479–93. doi: 10.1681/ASN.2016010045 PMC5280013 27444565

[40] McGuinness D Leierer J Shapter O Mohammed S Gingell-Littlejohn M Kingsmore DB. Identification of molecular markers of delayed graft function based on the regulation of biological ageing. PLoS One (2016) 11:e0146378. doi: 10.1371/journal.pone.0146378 26734715 PMC4703336

[41] Wilflingseder J Sunzenauer J Toronyi E Heinzel A Kainz A Mayer B. Molecular pathogenesis of post-transplant acute kidney injury: assessment of whole-genome mRNA and miRNA profiles. PLoS One (2014) 9:e104164. doi: 10.1371/journal.pone.0104164 25093671 PMC4122455

[42] Glowacki F Savary G Gnemmi V Buob D van der Hauwaert C Lo-Guidice JM. Increased circulating miR-21 levels are associated with kidney fibrosis. PLoS One (2013) 8:e58014. doi: 10.1371/journal.pone.0058014 23469132 PMC3585177

[43] Ben-Dov IZ Muthukumar T Morozov P Mueller FB Tuschl T Suthanthiran M. MicroRNA sequence profiles of human kidney allografts with or without tubulointerstitial fibrosis. Transplantation (2012) 94:1086–94. doi: 10.1097/TP.0b013e3182751efd PMC3541003 23131772

[44] Svenningsen P Sabaratnam R Jensen BL. Urinary extracellular vesicles: origin, role as intercellular messengers and biomarkers; efficient sorting and potential treatment options. Acta Physiol (Oxf) (2020) 228:e13346. doi: 10.1111/apha.13346 31334916

[45] Ghai V Wu X Bheda-Malge A Argyropoulos CP Bernardo JF Orchard T. Genome-wide profiling of urinary extracellular vesicle microRNAs associated with diabetic nephropathy in type 1 diabetes. Kidney Int Rep (2018) 3:555–72. doi: 10.1016/j.ekir.2017.11.019 PMC5976846 29854963

[46] Erdbrugger U Le TH. Extracellular vesicles in renal diseases: more than novel biomarkers? J Am Soc Nephrol (2016) 27:12–26. doi: 10.1681/ASN.2015010074 26251351 PMC4696584

[47] Salih M Zietse R Hoorn EJ. Urinary extracellular vesicles and the kidney: biomarkers and beyond. Am J Physiol Renal Physiol (2014) 306:F1251–9. doi: 10.1152/ajprenal.00128.2014 24694589

[48] Sole C Moline T Vidal M Ordi-Ros J Cortes-Hernandez J. An exosomal urinary miRNA signature for early diagnosis of renal fibrosis in lupus nephritis. Cells (2019) 8. doi: 10.3390/cells8080773 PMC6721515 31349698

[49] Schuierer S Carbone W Knehr J Petitjean V Fernandez A Sultan M. A comprehensive assessment of RNA-seq protocols for degraded and low-quantity samples. BMC Genomics (2017) 18:442. doi: 10.1186/s12864-017-3827-y 28583074 PMC5460543

[50] Garcia-Contreras M Shah SH Tamayo A Robbins PD Golberg RB Mendez AJ. Plasma-derived exosome characterization reveals a distinct microRNA signature in long duration type 1 diabetes. Sci Rep (2017) 7:5998. doi: 10.1038/s41598-017-05787-y 28729721 PMC5519761

[51] Val S Jeong S Poley M Krueger A Nino G Brown K. Purification and characterization of microRNAs within middle ear fluid exosomes: implication in otitis media pathophysiology. Pediatr Res (2017) 81:911–8. doi: 10.1038/pr.2017.25 PMC8900972 28157838

[52] Pfeffer SR Grossmann KF Cassidy PB Yang CH Fan M Kopelovich L. Detection of exosomal miRNAs in the plasma of melanoma patients. J Clin Med (2015) 4:2012–27. doi: 10.3390/jcm4121957 PMC4693157 26694476

[53] Armstrong DA Green BB Seigne JD Schned AR Marsit CJ. MicroRNA molecular profiling from matched tumor and bio-fluids in bladder cancer. Mol Cancer (2015) 14:194. doi: 10.1186/s12943-015-0466-2 26576778 PMC4650939

[54] Kurian SM Whisenant T Mas V Heilman R Abecassis M Salomon DR. Biomarker guidelines for high-dimensional genomic studies in transplantation: adding method to the madness. Transplantation (2017) 101:457–63. doi: 10.1097/TP.0000000000001622 28212255

[55] Seo JW Lee YH Tae DH Park SH Moon JY Jeong KH. Non-invasive diagnosis for acute rejection using urinary mRNA signature reflecting allograft status in kidney transplantation. Front Immunol (2021) 12:656632. doi: 10.3389/fimmu.2021.656632 34177898 PMC8222723

[56] Li Z Deng X Kang Z Wang Y Xia T Ding N. Elevation of miR-21, through targeting MKK3, may be involved in ischemia pretreatment protection from ischemia-reperfusion induced kidney injury. J Nephrol (2016) 29:27–36. doi: 10.1007/s40620-015-0217-x 26149640

[57] Liu E Lv L Zhan Y Ma Y Feng J He Y. METTL3/N6-methyladenosine/ miR-21-5p promotes obstructive renal fibrosis by regulating inflammation through SPRY1/ERK/NF-kappaB pathway activation. J Cell Mol Med (2021) 25:7660–74. doi: 10.1111/jcmm.16603 PMC8358893 34164910

[58] Sun L Xu T Chen Y Qu W Sun D Song X. Pioglitazone attenuates kidney fibrosis *via* miR-21-5p modulation. Life Sci (2019) 232:116609. doi: 10.1016/j.lfs.2019.116609 31254585

[59] Xu BY Meng SJ Shi SF Liu LJ Lv JC Zhu L. MicroRNA-21-5p participates in IgA nephropathy by driving T helper cell polarization. J Nephrol (2020) 33:551–60. doi: 10.1007/s40620-019-00682-3 31863364

[60] Zhou B Wu LL Zheng F Wu N Chen AD Zhou H. miR-31-5p promotes oxidative stress and vascular smooth muscle cell migration in spontaneously hypertensive rats *via* inhibiting FNDC5 expression. Biomedicines (2021) 9. doi: 10.3390/biomedicines9081009 PMC8393189 34440213

[61] Fang R Cao X Zhu Y Chen Q. Hsa_circ_0037128 aggravates high glucose-induced podocytes injury in diabetic nephropathy through mediating miR-31-5p/KLF9. Autoimmunity (2022) 55:254–63. doi: 10.1080/08916934.2022.2037128 35285770

[62] Liu P Wang S Wang G Zhao M Du F Li K. Macrophage-derived exosomal miR-4532 promotes endothelial cells injury by targeting SP1 and NF-kappaB P65 signalling activation. J Cell Mol Med (2022) 26:5165–80. doi: 10.1111/jcmm.17541 PMC9575109 36071548

[63] Zhao C Du F Zhao Y Wang S Qi L. Acute myeloid leukemia cells secrete microRNA-4532-containing exosomes to mediate normal hematopoiesis in hematopoietic stem cells by activating the LDOC1-dependent STAT3 signaling pathway. Stem Cell Res Ther (2019) 10:384. doi: 10.1186/s13287-019-1475-7 31842997 PMC6915875

[64] Feng F Zhu X Wang C Chen L Cao W Liu Y. Downregulation of hypermethylated in cancer-1 by miR-4532 promotes adriamycin resistance in breast cancer cells. Cancer Cell Int (2018) 18:127. doi: 10.1186/s12935-018-0616-x 30202238 PMC6123967

[65] Szeto CC Ng JK Fung WW Luk CC Wang G Chow KM. Kidney microRNA-21 expression and kidney function in IgA nephropathy. Kidney Med (2021) 3:76–82.e1. doi: 10.1016/j.xkme.2020.11.009 33604541 PMC7873829

[66] Zang J Maxwell AP Simpson DA McKay GJ. Differential expression of urinary exosomal MicroRNAs miR-21-5p and miR-30b-5p in individuals with diabetic kidney disease. Sci Rep (2019) 9:10900. doi: 10.1038/s41598-019-47504-x 31358876 PMC6662907

[67] Zununi Vahed S Poursadegh Zonouzi A Ghanbarian H Ghojazadeh M Samadi N Omidi Y. Differential expression of circulating miR-21, miR-142-3p and miR-155 in renal transplant recipients with impaired graft function. Int Urol Nephrol (2017) 49:1681–9. doi: 10.1007/s11255-017-1602-2 28455659

[68] Pavkovic M Robinson-Cohen C Chua AS Nicoara O Cardenas-Gonzalez M Bijol V. Detection of drug-induced acute kidney injury in humans using urinary KIM-1, miR-21, -200c, and -423. Toxicol Sci (2016) 152:205–13. doi: 10.1093/toxsci/kfw077 PMC5009468 27122240

[69] Du J Cao X Zou L Chen Y Guo J Chen Z. MicroRNA-21 and risk of severe acute kidney injury and poor outcomes after adult cardiac surgery. PloS One (2013) 8:e63390. doi: 10.1371/journal.pone.0063390 23717419 PMC3662667

[70] Saikumar J Hoffmann D Kim TM Gonzalez VR Zhang Q Goering PL. Expression, circulation, and excretion profile of microRNA-21, -155, and -18a following acute kidney injury. Toxicol Sci (2012) 129:256–67. doi: 10.1093/toxsci/kfs210 PMC3499041 22705808

[71] Cardenas-Gonzalez M Srivastava A Pavkovic M Bijol V Rennke HG Stillman IE. Identification, confirmation, and replication of novel urinary MicroRNA biomarkers in lupus nephritis and diabetic nephropathy. Clin Chem (2017) 63:1515–26. doi: 10.1373/clinchem.2017.274175 PMC5610914 28667184

[72] Monteiro MB Santos-Bezerra DP Pelaes TS Vaidya VS Correa-Giannella ML. MicroRNAs 1915-3p, 2861, and 4532 are associated with long-term renal function decline in type 1 diabetes. Clin Chem (2019) 65:1458–9. doi: 10.1373/clinchem.2019.307686 PMC7055663 31427290

[73] Zhang L Li H Yuan M Li D Sun C Wang G. Serum exosomal MicroRNAs as potential circulating biomarkers for endometriosis. Dis Markers (2020) 2020:2456340. doi: 10.1155/2020/2456340 32076458 PMC7008302

[74] Lu G Wong MS Xiong MZQ Leung CK Su XW Zhou JY. Circulating MicroRNAs in delayed cerebral infarction after aneurysmal subarachnoid hemorrhage. J Am Heart Assoc (2017) 6. doi: 10.1161/JAHA.116.005363 PMC5533026 28442458

[75] Bloom RD Bromberg JS Poggio ED Bunnapradist S Langone AJ Sood P. Cell-free DNA and active rejection in kidney allografts. J Am Soc Nephrol (2017) 28:2221–32. doi: 10.1681/ASN.2016091034 PMC5491290 28280140

