# Corrigendum: Development and validation of urinary exosomal microRNA biomarkers for the diagnosis of acute rejection in kidney transplant recipients

**DOI:** 10.3389/fimmu.2023.1234336

**Published:** 2023-06-13

**Authors:** Jung-Woo Seo, Yu Ho Lee, Dong Hyun Tae, Yang Gyun Kim, Ju-Young Moon, Su Woong Jung, Jin Sug Kim, Hyeon Seok Hwang, Kyung-Hwan Jeong, Hye Yun Jeong, So-Young Lee, Byung Ha Chung, Chan-Duck Kim, Jae Berm Park, Junhee Seok, Yeong Hoon Kim, Sang-Ho Lee

**Affiliations:** ^1^ Division of Nephrology, Department of Internal Medicine, Kyung Hee University, Seoul, Republic of Korea; ^2^ Research Laboratory, Medical Science Institute, Kyung Hee University Hospital at Gangdong, Seoul, Republic of Korea; ^3^ Division of Nephrology, Department of Internal Medicine, CHA Bundang Medical Center, CHA University, Seongnam, Republic of Korea; ^4^ School of Electrical Engineering, Korea University, Seoul, Republic of Korea; ^5^ Research Center, Division of Nephrology, Department of Internal Medicine, Seoul St. Mary’s Hospital, College of Medicine, The Catholic University of Korea, Seoul, Republic of Korea; ^6^ Division of Nephrology, Department of Internal Medicine, Kyungpook National University Hospital, Daegu, Republic of Korea; ^7^ Department of Surgery, Samsung Medical Center, Seoul, Republic of Korea; ^8^ Department of Internal Medicine, Inje University Busan Paik Hospital, Busan, Republic of Korea

**Keywords:** kidney transplantation, acute rejection, urine, exosome, microRNA

In the published article, there was an error in the Funding statement.

The funding statement for “National Research Foundation of Korea, funded by the Korean Government Ministry of Science and ICT (grant number 2018M3A9E8078807) should be changed to “Korean Health Technology R&D Project, Ministry of Health & Welfare (grant number HV22C019300)”.

The correct Funding statement appears below.

**Funding**

This work was supported by the National Research Foundation of Korea, funded by the Korean Government Ministry of Science and ICT (grant number RS-2023-00213976), the Korean Health Technology R&D Project, Ministry of Health & Welfare (grant number HV22C019300), and a grant from Research year of Inje University in 2020.

The authors apologize for this error and state that this does not change the scientific conclusions of the article in any way. The original article has been updated.

