# Supplementary material for: Development and validation of urinary exosomal microRNA biomarkers for the diagnosis of acute rejection in kidney transplant recipients
Source: Front Immunol. 2023 May 9;14:1190576. doi: 10.3389/fimmu.2023.1190576 (PMC10203902; doi:10.3389/fimmu.2023.1190576)
Supplement: Supplementary file 1 [file DataSheet_1.docx]

Supplementary Material

Development and validation of urinary exosomal microRNA biomarkers for the diagnosis of acute rejection in kidney transplant recipients

Jung-Woo Seo^1,2*^, Yu Ho Lee^3*^, Dong Hyun Tae^4^, Yang Gyun Kim^1^, Ju-Young Moon^1^, Su Woong Jung^1^, Jin Sug Kim^1^, Hyeon Seok Hwang^1^, Kyung-Hwan Jeong^1^, Hye Yun Jeong^3^, So-Young Lee^3^, Byung Ha Chung^5^, Chan-Duck Kim^6^, Jae Berm Park^7^, Junhee Seok^4^, Yeong Hoon Kim^8†^, and Sang-Ho Lee^1,2†^

^1^Division of Nephrology, Department of Internal Medicine, Kyung Hee University, Seoul, South Korea

^2^Research Laboratory, Medical Science Institute, Kyung Hee University Hospital at Gangdong, Seoul, South Korea

^3^Division of Nephrology, Department of Internal Medicine, CHA Bundang medical center, CHA University, Seongnam, Korea

^4^School of Electrical Engineering, Korea University, Seoul, Korea

^5^Research Center, Division of Nephrology, Department of Internal Medicine, Seoul St. Mary’s Hospital, College of Medicine, The Catholic University of Korea, Seoul, Korea

^6^Division of Nephrology, Department of Internal Medicine, Kyungpook National University Hospital, Daegu, Korea

^7^Department of Surgery, Samsung Medical center, Seoul, Korea

^8^Department of Internal Medicine, Inje University Busan Paik Hospital, Busan, Korea

*** Contributed equally to this work as co-first authors**

^†^ **Contributed equally to this work as co-corresponding authors**

**Correspondence to:**

**Sang-Ho Lee, M.D., Ph.D.**

Division of Nephrology, Department of Internal Medicine, Kyung Hee University, Seoul

E-mail: [lshkidney@khu.ac.kr](mailto:lshkidney@khu.ac.kr)

**Yeong Hoon Kim, M.D., Ph.D.**

Department of Internal Medicine, Inje University Busan Paik Hospital, Busan, Korea

E-mail: yeonghnl@inje.ac.kr

**Supplementary Figure 1.** Heatmap of urinary exosomal microRNAs differentially expressed in acute rejection group

**
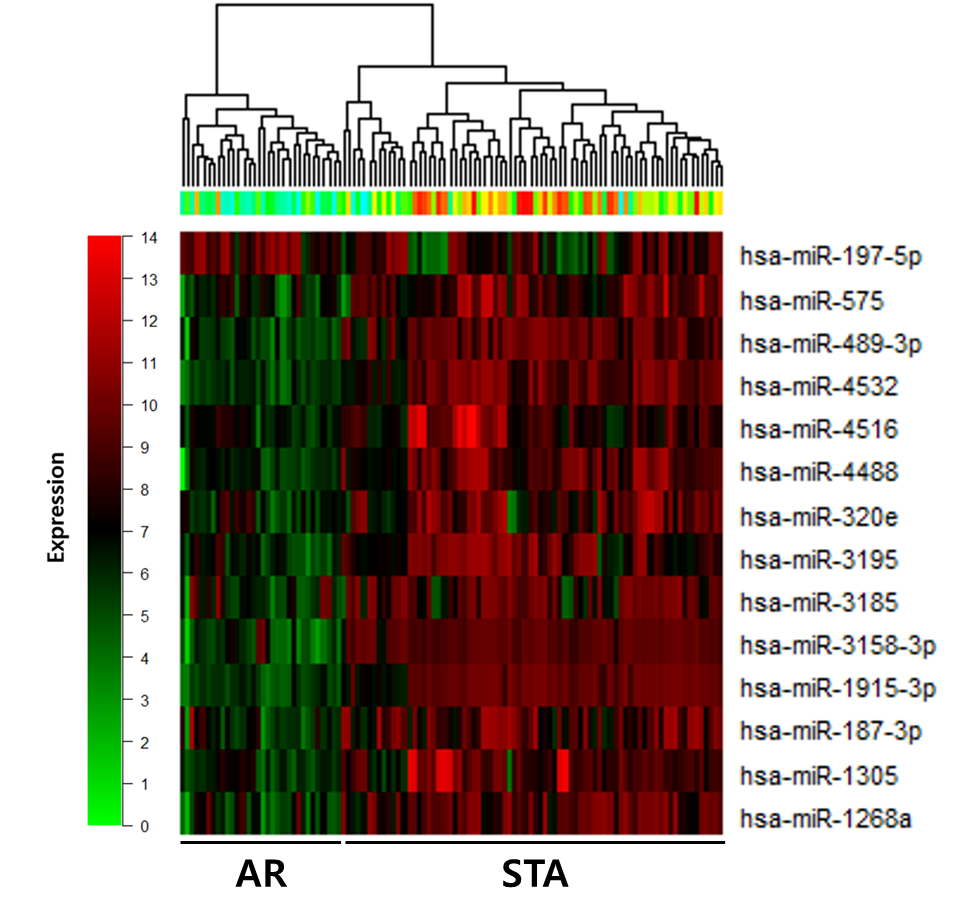
**

Shown are the hierarchical clustering of 14 urinary exosomal microRNAs deferentially expressed in patients with acute rejection (AR) compared with those maintaining stable graft function (STA) identified by Nanostring analysis. Most microRNAs (13/14, 92.9%) were down regulated while only one microRNA (hsa-miR-197-5p) were upregulated in acute rejection group.

Abbreviations: AR, acute rejection; STA, stable graft function.

**Supplementary Figure 2.** Expressions of acute rejection-specific microRNA candidates in urinary exosomes according to diagnostic groups

**
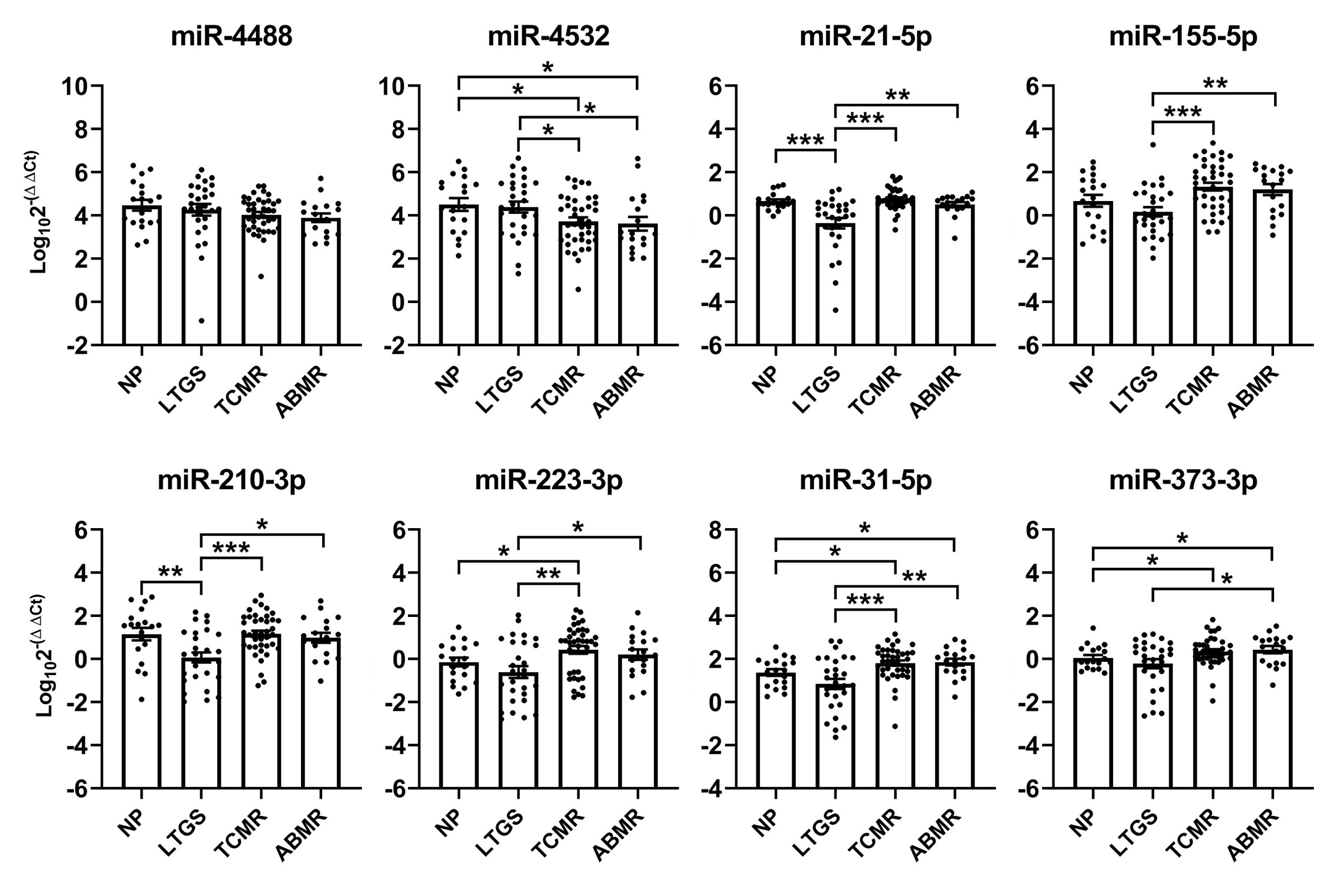
**

Each microRNA is normalized by the level of hsa-miR-16-5p and is expressed as ddCt (delta delta cycle threshold) after log transformation.

Abbreviations: NP: normal pathology on biopsy; LTGS, long-term good survival; TCMR, acute T cell-mediated rejection; ABMR, acute antibody-mediated rejection). ^*^*p*<0.05, ^**^*p*<0.01, ^***^*p*<0.001.

**Supplementary Figure 3.** Expressions of acute rejection-specific microRNA candidates in urinary exosomes from antibody-mediated rejection group according to the presence of donor specific antibodies at the time of biopsy


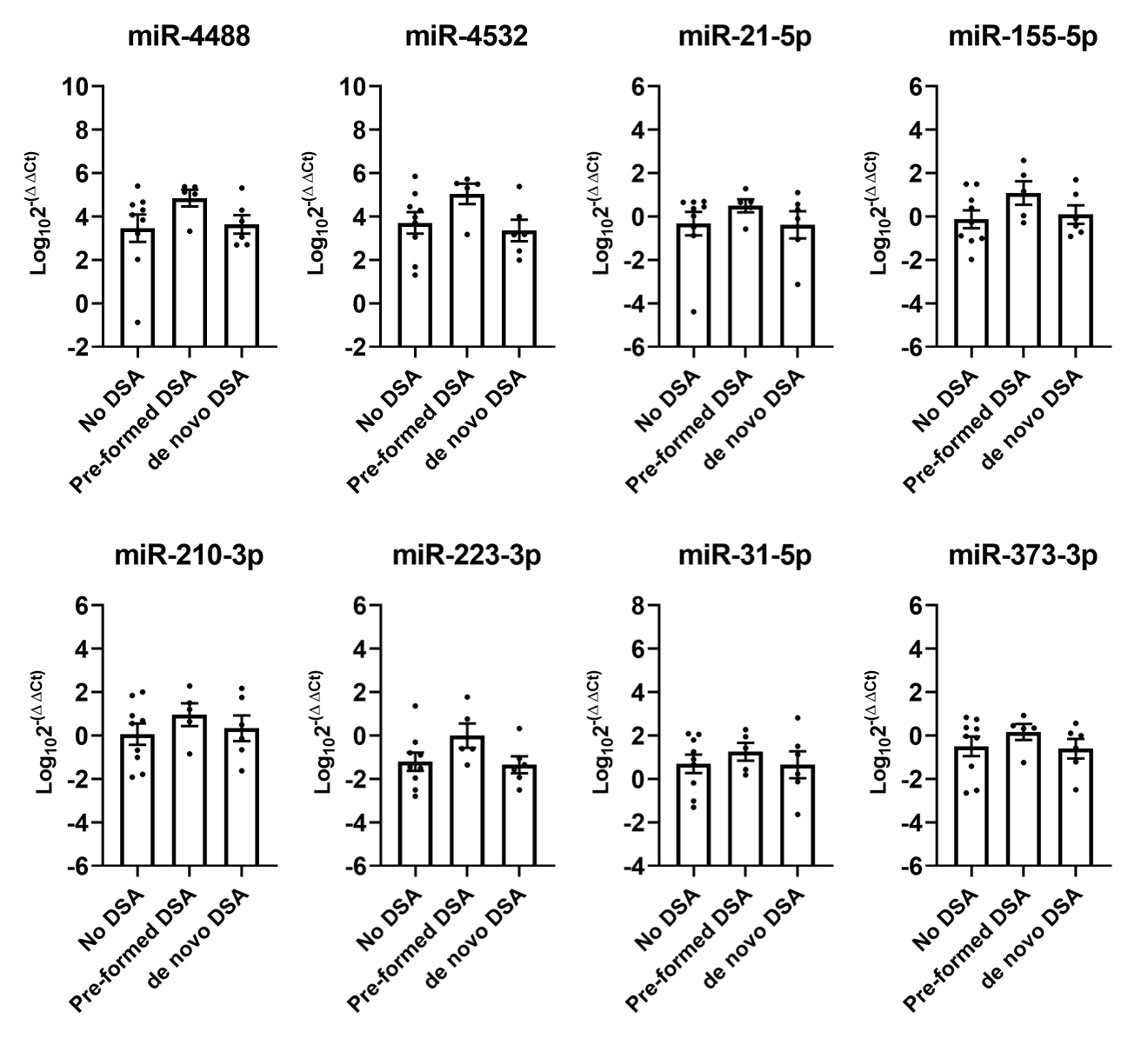


Each microRNA is normalized by the level of hsa-miR-16-5p and is expressed as ddCt (delta delta cycle threshold) after log transformation.

Abbreviations: DSA, donor specific antibody.

**Supplementary Figure 4.** Expressions of acute rejection-specific microRNAs in urinary exosomes of validation set


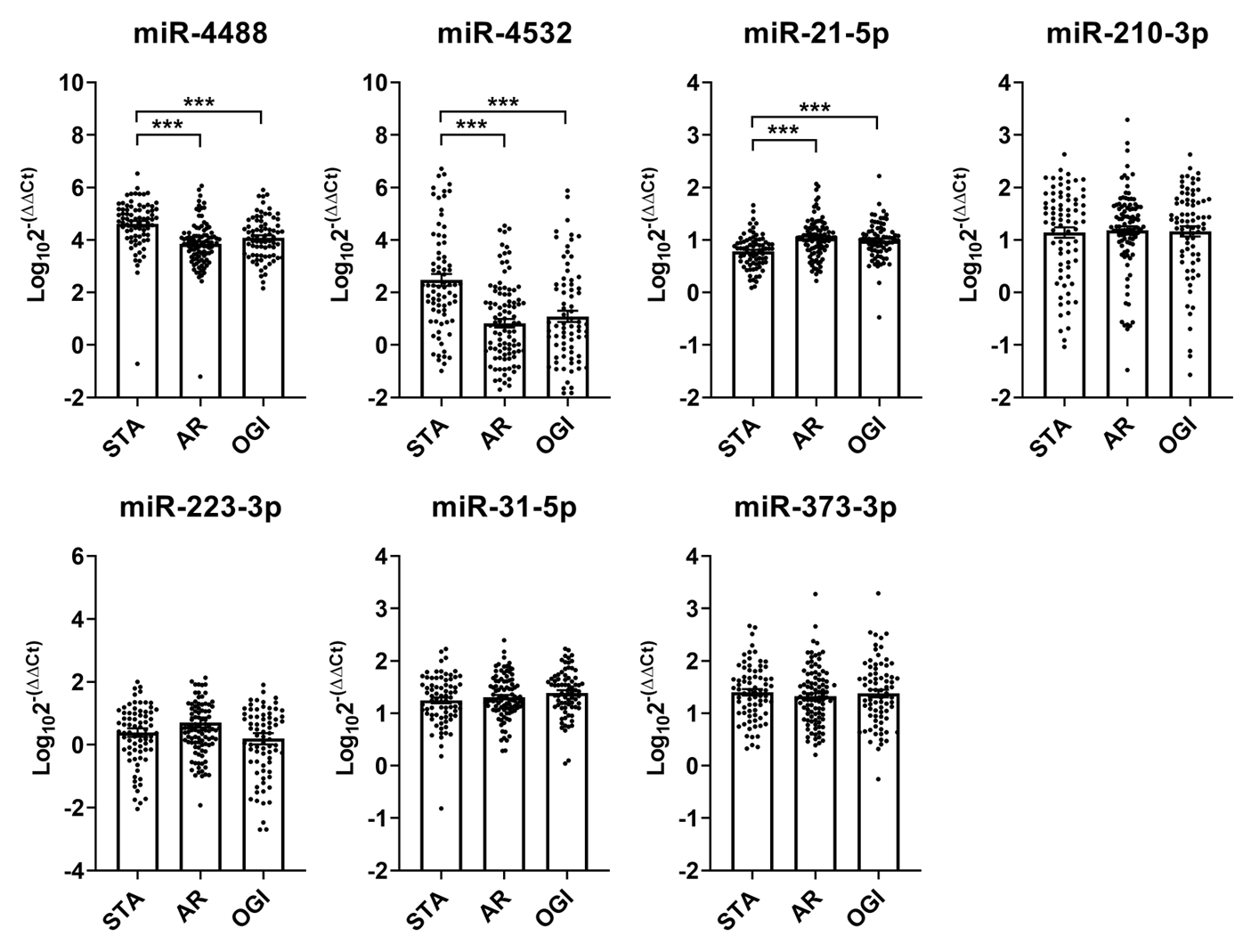


Shown are the levels of AR-specific urinary exosomal microRNAs in validation set. hsa-miR-155-5p, one of the AR-specific urinary exosomal microRNAs identified in discovery set, is not shown here because it failed to pass quality control (not measured in more than 20% of samples). Each microRNA level was normalized by hsa-miR-16-5p and expressed as ddCt (delta delta cycle threshold) after log transformation.

OGI includes acute tubular necrosis, glomerulopathy, calcineurin inhibitor toxicity, and BK virus associated nephropathy.

Abbreviations: STA: stable graft function; LTGS, AR, acute rejection; OGI, other graft injuries. ^***^*p*<0.001.

**Supplementary Table 1.** Baseline characteristics and clinical parameters of enrolled patients according to clinicopathologic diagnosis

|  | Discovery set (n=108) | | | | | Validation set (n=260) | | | | | | |  |
| --- | --- | --- | --- | --- | --- | --- | --- | --- | --- | --- | --- | --- | --- |
|  | NP  (n=19) | LTGS  (n=29) | TCMR  (n=40) | aABMR  (n=20) | *p* | NP  (n=50) | LTGS  (n=30) | TCMR  (n=50) | aABMR  (n=30) | cABMR  (n=20) | OGI*  (n=80) | *p* | |
| Age (years) | 47.3±9.8 | 57.4±9.5 | 47.6±12.0 | 47.5±9.1 | 0.001 | 45.6±13.4 | 50.9±8.5 | 46.6±12.4 | 48.5±9.0 | 47.9±12.3 | 47.4±13.1 | 0.524 | |
| Sex (Male, %) | 11 (57.9) | 8 (27.6) | 25 (62.5) | 14 (70.0) | 0.009 | 27 (54.0) | 15 (50.0) | 38 (76.0) | 19 (63.3) | 12 (60.0) | 60 (75.0) | 0.033 | |
| Deceased donor KT (n, %) | 9 (47.4) | 4 (13.8) | 24 (60.0) | 3 (15.0) | <0.001 | 16 (32.0) | 4 (13.3) | 22 (44.0) | 9 (30.0) | 8 (40.0) | 35 (43.8) | 0.050 | |
| Duration after KT (year) | 0.5±0.6 | 16.2±5.7 | 1.1±1.4 | 2.3±4.1 | <0.001 | 0.4±1.0 | 14.6±4.1 | 1.9±2.2 | 5.9±4.8 | 8.1±4.5 | 2.7±4.5 | <0.001 | |
| ABO incompatible KT (n, %) | 2 (10.5) | 0 (0) | 6 (15.0) | 8 (40.0) | 0.488 | 4 (8.0) | 1 (3.3) | 5 (10.0) | 2 (6.7) | 1 (5.0) | 5 (6.2) | 0.901 | |
| HLA mismatching (n) | 3.1±1.8 | 2.1±1.3 | 3.5±1.4 | 4.1±1.4 | 0.001 | 2.9±1.7 | 3.1±1.5 | 3.3±1.6 | 3.3±1.6 | 3.0±1.4 | 3.4±1.8 | 0.710 | |
| Donor specific antibody (n, %) | - | - | - |  | - | - | - | - |  |  | - | - | |
| Negative |  |  |  | 5 (25.0) |  |  |  |  | 15 (50.0) | 13 (65.0) |  |  |  |
| Pre-formed |  |  |  | 9 (45.0) |  |  |  |  | 7 (23.3) | 3 (15.0) |  |  |  |
| De novo |  |  |  | 6 (30.0) |  |  |  |  | 8 (26.7) | 4 (20.0) |  |  |  |
| eGFR (ml/min/1.73m^2^) | 73.3±15.6 | 67.3±10.1 | 37.8±23.7 | 37.2±18.7 | <0.001 | 80.5±22.9 | 71.8±14.8 | 34.8±14.5 | 32.9±15.1 | 30.8±18.4 | 39.4±17.0 | <0.001 | |
| Urine PCR (mg/gCr) | 88±42 | 224±360 | 796±1230 | 1587±2014 | 0.002 | 228±423 | 243±241 | 984±1576 | 2162±2380 | 2111±1900 | 1245±1950 | <0.001 | |
| Induction immunosuppression |  |  |  |  | 0.084 |  |  |  |  |  |  | 0.115 | |
| Basiliximab (n, %) | 15 (78.9) | 25 (86.2) | 27 (67.5) | 11 (55.0) |  | 35 (70.0) | 30 (100) | 38 (76.0) | 29 (96.7) | 20 (100) | 63 (78.8) |  |  |
| Anti-thymocyte globulin (n, %) | 4 (21.1) | 4 (13.8) | 13 (32.5) | 9 (45.0) |  | 15 (30.0) | 0 (0) | 12 (24.0) | 1 (3.3) | 0 (0) | 17 (21.2) |  |  |
| Maintenance immunosuppression |  |  |  |  |  |  |  |  |  |  |  |  | |
| Steroid (n, %) | 17 (89.5) | 13 (44.8) | 34 (85.0) | 18 (90.0) | <0.001 | 49 (98.0) | 21 (70.0) | 44 (88.0) | 29 (96.7) | 16 (80.0) | 74 (92.5) | 0.001 | |
| Calcineurin inhibitor (n, %) | 19 (100) | 25 (86.2) | 37 (92.5) | 20 (100) | 0.128 | 49 (98.0) | 28 (93.3) | 46 (92.0) | 27 (93.3) | 19 (95.0) | 77 (96.2) | 0.613 | |
| Mycophenolate mofetil (n, %) | 19 (100) | 12 (41.4) | 26 (65.0) | 19 (95.0) | 0.001 | 48 (96.0) | 23 (76.7) | 43 (86.0) | 27 (90.0) | 14 (70.0) | 64 (80.0) | 0.042 | |
| mTOR inhibitor (n, %) | 2 (10.5) | 3 (10.3) | 4 (10.0) | 0 (0) | 0.766 | 3 (6.0) | 2 (6.7) | 2 (4.0) | 3 (10.0) | 2 (10.0) | 5 (6.2) | 0.908 | |
| Donor age (years) | 40.5±15.1 | 29.5±14.0 | 47.4±13.6 | 43.4±10.5 | <0.001 | 44.7±14.2 | 33.5±12.9 | 47.7±14.8 | 41.8±23.4 | 45.1±13.7 | 49.3±12.5 | 0.001 | |
| Donor sex (Male, %) | 12 (63.2) | 18 (62.1) | 26 (65.0) | 9 (45.0) | 0.489 | 29 (58.0) | 15 (50.0) | 25 (50.0) | 12 (40.0) | 11 (55.0) | 38 (47.5) | 0.756 | |

**Abbreviations**: NP, normal pathology; LTGS, long term graft survival; TCMR, acute T cell-mediated rejection; aABMR, acute antibody-mediated rejection; cABMR, chronic active antibody-mediated rejection; OGI, other graft injuries; KT, kidney transplantation; eGFR, estimated glomerular filtration rate; PCR, protein-to-creatinine ratio.

Data are expressed as mean±standard deviation or number of patients (percentage).

* OGI includes acute tubular necrosis (n=20), de novo or recurrent glomerulonephritis (n=20), and interstitial fibrosis and tubular atrophy (n=20), and BK virus associated nephropathy (n=20).
